# Electroconvulsive therapy use in adolescents: a systematic review

**DOI:** 10.1186/1744-859X-12-17

**Published:** 2013-05-30

**Authors:** Nádia NR Lima, Vânia B Nascimento, Jorge AC Peixoto, Marcial M Moreira, Modesto LR Neto, José C Almeida, Carlos AC Vasconcelos, Saulo A Teixeira, Jucier G Júnior, Francisco TC Junior, Diego DM Guimarães, Aline Q Brasil, Jesus S Cartaxo, Marco Akerman, Alberto OA Reis

**Affiliations:** 1 Health Sciences Postgraduate Program, Faculty of Medicine, ABC Foundation, Santo André, São Paulo 09060-650, Brazil; 2 Research Group CNPq/UFC: Information Technology, Communication, Narrativity, Society and Plural Identities, Federal University of Ceará (UFC), Juazeiro do Norte, Ceará 63.048-060, Brazil; 3 Federal University of Campina Grande (UFCG), Cajazeiras, Paraíba 58900-000, Brazil; 4 Neuropsychiatry and Behavioral Sciences Postgraduate Program, Federal University of Pernambuco (UFPE), Recife, Pernambuco 50.670-420, Brazil; 5 Faculty of Medicine, Federal University of Ceará (UFC), Barbalha, Ceará 63180-000, Brazil; 6 Public Health Faculty, University of São Paulo (USP), São Paulo 01246-904, Brazil

## Abstract

**Background:**

Considered as a moment of psychological vulnerability, adolescence is remarkably a risky period for the development of psychopathologies, when the choice of the correct therapeutic approach is crucial for achieving remission. One of the researched therapies in this case is electroconvulsive therapy (ECT). The present study reviews the recent and classical aspects regarding ECT use in adolescents.

**Methods:**

Systematic review, performed in November 2012, conformed to the PRISMA statement.

**Results:**

From the 212 retrieved articles, only 39 were included in the final sample. The reviewed studies bring indications of ECT use in adolescents, evaluate the efficiency of this therapy regarding remission, and explore the potential risks and complications of the procedure.

**Conclusions:**

ECT use in adolescents is considered a highly efficient option for treating several psychiatric disorders, achieving high remission rates, and presenting few and relatively benign adverse effects. Risks can be mitigated by the correct use of the technique and are considered minimal when compared to the efficiency of ECT in treating psychopathologies.

## Background

Adolescence is a period of deep emotional and physical changes. This moment of psychological vulnerability favors the development of serious and potentially damaging psychopathologies [1]. Adolescents present higher rates of self-destruction impulses and are more commonly refractory to traditional medications. In addition, major depression affects near 8% of adolescents, more commonly during puberty. Forty percent of them present recurrent attacks, and a third will experience at least one suicide attempt [2]. In these cases, the choice of the correct therapeutic modalities is crucial for achieving the remission of symptoms.

The introduction of electroconvulsive therapy (ECT) for the treatment of serious mental disturbs, such as major depression and bipolar disorder, was one of most impacting revolutions of psychiatry. Convulsive therapy was introduced to psychiatric practice in 1934, by Hungarian neuropsychiatrist László Meduna [3]. In his autobiography [4], Meduna described L. Zoltán's case, his first patient subjected to convulsive therapy [3,4]. L. Zoltán had a 4-year condition of catatonic schizophrenia that was considered hopeless but after having received several sessions of camphor-induced convulsive therapy [4,5], achieved full remission [3]. Later on, in 1938, Bini and Cerletti reported the first electroconvulsive procedure, suggesting the use of electricity after observing the clinical success and difficulties of seizure inductions with metrazol [6].

Due to controversies regarding its effectiveness and safety, for a long time, ECT had a bad reputation and was considered untrustworthy. Only in the 1970s, the American Task Force Report on ECT proved the effectiveness of ECT use, especially in the depressed phase of bipolar disorder and the ‘involutional melancholia’ [7].

Brazilian Federal Council of Medicine, in its Resolution n. 1640, published in July 2002, regulates the use of electroconvulsive therapy in Brazil. Still, it prohibited the use of ECT in patients below 16 years of age, unless in exceptional circumstances [8].

Along these lines, a key question addressed by this study is whether ECT is useful for therapeutic purposes in adolescents. Taking this into consideration, this study aimed at reviewing the recent and classical aspects regarding electroconvulsive therapy and its application for adolescents; we performed a detailed, systematic review of medical literature, of which resulted in this article.

## Methods

We performed, in November 2012, a qualitative systematic review of articles about the role of electroconvulsive therapy use in adolescents published in previously chosen electronic databases. The qualitative approach was chosen because quantitative methods, such as meta-analysis, show that (a) the necessary information in order to calculate result size is not available and may limit this analysis to a small subset of studies and (b) age intervals regarding adolescence vary greatly among studies included in the sample, making it difficult to adequately compare and to do the proper statistical analysis.

The methods used to identify, select and appraise relevant research, and to collect and analyze data from the studies we reviewed were in agreement with the PRISMA protocol for systematic reviews and meta-analyses [9]. According to The Cochrane Collaboration, which published the PRISMA guidelines, reporting medical research in systematic reviews must be based in information that composes the acronym ‘PICOS’ (patient, intervention, comparator, outcome, study design). For this article, ‘P’ represented adolescents, ‘I’ was electroconvulsive therapy, ‘C’ was absent or only-pharmacological therapy, ‘O’ was symptoms remission and ‘S’ was case reports, series of cases, literature reviews, cross-sectional studies, exploratory field research and prospective/retrospective cohorts.

In order to set a parameter for limiting the age group covered by the present review, the definition of ‘adolescence’ adopted in this study was from Medical Subject Headings MeSH), the National Library of Medicine's controlled vocabulary thesaurus used for indexing articles for PubMed. Thus, when used in this study, the term ‘adolescent’ refers to a person from 13 to 18 years of age and consequently, the term ‘adolescence’ refers to the state or time of being 13 to 18 years of age.

Then, each paper in the sample was read entirely, and data elements were then extracted and entered into a matrix that included authors, publication year, description of the study sample and main findings, as well as a matrix that included the PICOS acronym of the selected articles. Some of the studies dealt not only with ECT use in adolescents but also in children and adults; because the focus of this study was on ECT use in adolescents, children- or adult-related results were not recorded or analyzed for this study.

Finally, so as to provide a better analysis, the next phase involved grouping, for heuristic reasons, results regarding the studied subject in four themes: (1) indications for ECT use in adolescents; (2) electroconvulsive therapy - treatment parameters; (3) efficiency of ECT, associations with other therapies and comparison between techniques; and (4) side effects, risks, and complications of ECT for adolescents.

### Literature review

Three medical databases were surveyed: PMC (United States National Library of Medicine), LILACS (Literatura Latino-Americana e do Caribe em Ciências da Saúde), and SciELO (Scientific Electronic Library Online). The keywords used in all searches were all valid MeSH (NLM/NIH) terms: ‘electroconvulsive therapy’, ‘electroshock’, ‘ECT’, ‘adolescence’, ‘adolescent’, ‘treatment’, ‘depression’, ‘schizophrenia’, and ‘bipolar disorder’.

### Eligibility criteria

Selected articles were according to the following inclusion criteria: (1) manuscripts written in English, Portuguese, Spanish, or French; (2) case reports, series of cases, case-controls, literature reviews, cross-sectional studies, exploratory field research, and prospective and retrospective cohort studies; (3) studies regarding the use of electroconvulsive therapy in adolescents, provided they attended at least three of the five previously mentioned PICOS criteria: adolescent, ECT, absent or only drugs, symptoms remission, and study design. Studies assessing other conditions, editorials, and letters to the editor were excluded.

## Results

Initially, the aforementioned search strategies resulted in 212 references. After assessing the title and abstract of the retrieved citations for eligibility based on the inclusion criteria, 139 articles were excluded after applying criteria 1 and 2, and 40 manuscripts were excluded after applying criterion 3.

**Figure 1:**
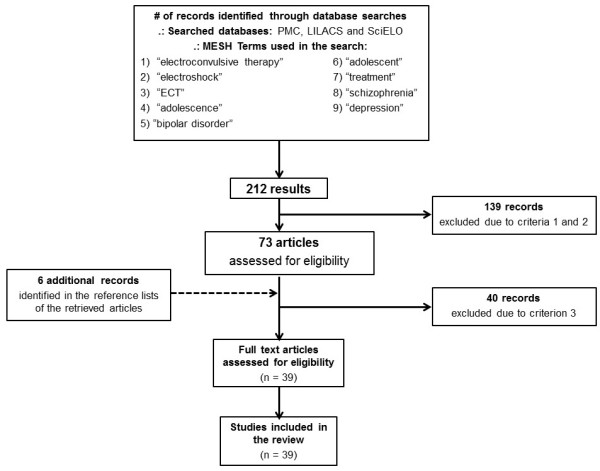
Flow chart showing study selection for the review.

At this moment, six additional articles were included in the sample [2-5,9,10], identified through the reference lists of the previously retrieved articles and considering their importance to the field of study. Finally, 39 articles were further selected and included in the final sample (Figure 1).

Table 1 provides an overview of all studies included in the final sample, synthesizing the studies' characteristics, with reference to participants, interventions, comparisons, outcomes, and study design (PICOS), according to PRISMA statement.

**Table 1:** Characteristics (PICOS) of the articles included in the review

**Author (publication year)**	**Patient**	**Intervention**	**Comparator**	**Outcome**	**Study design**
Gedge et al. [11] (2012)	Age 18 or older	ECT and rTMS	Absent	Serum BDNF may not be a biomarker of ECT or rTMS treatment response	Prospective cohort
Garg et al. [12] (2011)	Age 16 to 65	ECT	Absent	Yes	Prospective cohort
Rolim-neto et al. [2] (2011)	Children and adolescents	-	Absent	Child depression	Literature review
Shoirah and Hamoda [13] (2011)	Adolescents	ECT	Absent	-	Literature review
Wachtel et al. [6] (2011)	Children and adolescents	ECT	Absent	Efficacy rate of 63% for depression	Literature review
Baeza et al. [7] (2010)	Age 13 to 17	Evaluation before, after and 6 months after ECT	Absent	ECT is safe and effective treatment for SSD	Retrospective cohort
Consoli et al. [14] (2010)	Children and adolescents	ECT	Absent	-	Literature review
Antunes et al. [15] (2009)	-	ECT	Absent	50 to 80%	Literature review
Hazell [1] (2009)	Children and adolescents	Various	Absent	No article about ECT met the including criteria	Systematic review
Moher et al. [9] (2009)	-	-	-	PRISMA statement	Review
Baghai and Moller [16] (2008)	-	ECT	Absent	ECT is treatment proven to be a highly effective treatment option depression	Literature review
Feliu et al. [17] (2008)	Age 19 to 96	ECT	Absent	Yes	Series of cases
Lévy-Rueff et al. [18] (2008)	Age 30 to 67	ECT	Absent	Moderate efficacy of M-ECT on schizophrenia	Retrospective cohort
Arshad et al. [19] (2007)	Age 16 and above	Questionnaire about ECT	Absent	Low acceptability of ECT	Cross-sectional study
Blaj et al. [20] (2007)	-	ECT	Absent	Questionnaire to psychiatrists	Exploratory field research
Datka et al. [21] (2007)	-	ECT	Only drugs	Only temporally affects working memory function	Prospective cohort
Prakash et al. [22] (2006)	Adults	ECT	Absent	Donepezil improves recovery time	Triple blind prospective cohort
Salleh et al. [23] (2006)	-	ECT	Absent	Efficacy and safety of ECT on depression treatment	Literature review
Stein et al. [24] (2006)	Children and adolescents	ECT	Absent	-	Literature review
Zaw [25] (2006)	Children and adolescents	ECT	Absent	-	Literature review
Sienaert et al. [26] (2005)	Adults and aged	ECT	Absent	ECT patients have memory complaints, but it is not related to satisfaction with treatment	Exploratory field research
Ghaziuddin et al. [27] (2004)	Adolescents	ECT	Absent	Mood disorders have a high rate of response (75% to 100%)	Literature review
Segal et al. [28] (2004)	Age 13	ECT	Absent	Full symptom resolution	Case report
Bloch et al. [29] (2001)	Age 13 to 19	ECT	Absent	58% remission	Retrospective cohort
Daly et al. [30] (2001)	Average group age of 58.5 and 55.8 years old	ECT	Absent	Yes	Prospective cohort
Cohen et al. [31] (2000)	Adolescents aged less than 19 years old	ECT	Only drugs	Cognitive functions similar to non-ECT	Retrospective cohort
Ghaziuddin et al. [32] (1999)	Age 16	ECT	Absent	Clinical improvement, but no significant change in depression score	Case report
Thuppal and Fink [33] (1999)	Adolescents and adults	ECT	Absent	ECT successful after pharmacotherapy failure on mental retardation	Series of cases
Kutcher and Robertson [34] (1995)	Age 16 to 22	ECT	Absent	Yes	Prospective cohort
Calev [35] (1994)	-	ECT	-	Trends on ECT treatment	Literature review
Parmar [36] (1993)	Children and adolescents, psychiatrists	ECT	Absent	ECT was less useful in child and adolescent psychotic depression	Questionnaire
Schneeklot et al. [37] (1993)	Adolescents	ECT	Absent	ECT reduced or eliminated symptoms	Retrospective case review
APA [38] (1990)	Various	ECT	Absent	Yes	Literature review
Paillère-Martinot et al. [39] (1990)	Adolescents	ECT	Absent	Yes	Comparative study

## Discussion

The present study reviewed 39 articles that dealt with different aspects of the use of ECT and its application in adolescents. Table 2 summarizes the main findings regarding the use of ECT in adolescents, grouped in three different sections. We now proceed to analyze the main findings of the retrieved studies.

**Table 2:** Electroconvulsive therapy use in adolescents: main findings

**Authors**	**Publication year**	**Journal**	**Main findings**
**Indications for ECT use in adolescents**			
Wachtel et al. [6]	2011	*Medical Hypotheses*	The use of ECT in children and adolescents is appropriate for specific clinical indications and urge removal of impediments to ECT access in this population
Baeza et al. [7]	2010	*Journal of Child and Adolescent Psychopharmacology*	ECT is a safe and effective treatment for schizophrenia spectrum disorders in adolescent patients
Consoli et al. [14]	2010	*Journal of ECT*	Electroconvulsive therapy is the effective treatment for catatonia after high-dose benzodiazepine trials in youths
Hazell [1]	2009	*Clinical Evidence*	Electroconvulsive therapy is indicated for a severely obtunded child or adolescent with depression who may, for example, have prolonged psychotic symptoms, and fails to hydrate or maintain caloric intake
Baghai and Moller [16]	2008	*Dialogues in Clinical Neuroscience*	The safety and tolerability of ECT have been enhanced by the use of modified stimulation techniques and by the progress of modern anesthesia, representing a safe treatment that can be offered to all patients, especially after medication failure
Stein et al. [24]	2006	*Child & Adolescent Psychiatric Clinics of North America*	ECT is an effective therapy for severe and resistant depression, with relatively minimal adverse effects
Ghaziuddin et al. [27]	2004	*Journal of the American Academy of Child and Adolescent Psychiatry*	Eligible adolescents for ECT must meet three criteria: diagnosis (severe, persistent major depression or mania, with or without psychosis, schizoaffective disorder, schizophrenia, and others), severity of symptoms, and lack of treatment response
**Electroconvulsive therapy: treatment parameters**			
Shoirah and Hamoda [13]	2011	*Expert Review of Neurotherapeutics*	Although bilateral electrode placement may be more effective than unilateral placement for manic patients, unilateral electrode placement has been found to have equivalent results for other indications
Baeza et al. [7]	2010	*Journal of Child and Adolescent Psychopharmacology*	The mean duration of electroencephalogram seizures was 43.9 ± 16.9 s (range, 20 to 93), with significant differences between males and females
Antunes et al. [15]	2009	*Revista Brasileira de Psiquiatria*	Studies show that high-dose unilateral ECT (UL-ECT) has an equivalent efficacy to bifrontotemporal ECT; however, low-dose UL-ECT has lower efficacy
**Efficiency of ECT**, **associations with other therapies and comparison between techniques**			
Garg et al. [12]	2011	*Indian Journal of Medical Research*	Patients with treatment-resistant schizophrenia treated with ECT had an improvement in quality of life. All aspects of quality of life got better, except the social relations
Lévy-Rueff et al. [18]	2008	*Psychiatry Research*	Part of a clinical cohort was composed by adolescents. Maintenance ECT in association with pharmacological treatment presented good outcomes for refractory schizophrenia
Bloch et al. [29]	2001	*Journal of the American Academy of Child and Adolescent Psychiatry*	ECT was equally effective in adolescents and adults (58% of remission), but most of adolescents presented psychotic syndromes, instead of affective disorders in adults
Strober et al. [10]	1998	*Biological Psychiatry*	Adolescents aged 13 to 17 years with bipolar depression or major depressive disorder presented 60% of total and 40% of partial remission in a month follow-up
Kutcher and Robertson [34]	1995	*Journal of Child and Adolescent Psychopharmacology*	Patients who accepted ECT improved significantly compared to those who refused. The mean duration of hospital stay was reduced from 176 to 73.8 days
Schneekloth et al. [37]	1993	*Convulsive Therapy*	Retrospective study with a 65% response rate among adolescents between 13 and 18 years old
Paillère-Martinot et al. [39]	1990	*Encephale*	Patients aged 15 to 19 years with different diagnoses achieved 88% of response rate after typical ECT application
**Side effects**, **risks, and complications of ECT for adolescents**			
Feliu et al. [17]	2008	*Neuropsychiatric Disease and Treatment*	Relatively immediate and significant decreases in multiple areas of memory following ECT, compared with pre-ECT levels of functioning, including verbal memory for word lists, prose passages, and visual memory of geometric designs
Datka et al. [21]	2007	*Klinika Psychiatrii CM UJ.*	One day after first ECT, patient's working memory was slightly impaired. ECT treatment affects working memory function only temporally
Prakash et al. [22]	2006	*Journal of ECT*	ECT presents cognitive side effects especially in recent memory. The post-ECT recovery of various components of cognition was more rapid in patients using donepezil and compared with placebo
Cohen et al. [31]	2000	*The American Journal of Psychiatry*	After a 3.5-year follow-up, patients who received ECT presented similar memory functions to those of psychiatric controls. Poorer cognitive performance is related with greater psychopathology, not with the treatment
Ghazziudin et al. [32]	1999	*Journal of Child and Adolescent Psychopharmacology*	Comparison with pre-ECT and post-ECT tests resulted in significant impairments of concentration and attention, verbal and visual delayed recall, and verbal fluency. Second stage of post-ECT tests (mean, 8.5 months after ECT) showed complete recovery and return to pre-ECT functioning

### Indications for ECT use in adolescents

Although information on the subject is scarce [29], indications for ECT use in adolescents cover a wide range of psychological diseases. The American Academy of Child and Adolescent Psychiatry (AACAP) has issued a guideline titled ‘Practice Parameter For Use of Electroconvulsive Therapy With Adolescents,’ establishing that eligibility for ECT in adolescents involves meeting three criteria, as follows: (1) diagnosis, (2) severity of symptoms, and (3) lack of treatment response to appropriate psychopharmacological agents accompanied by other appropriate treatment modalities [27]. Regarding diagnosis, ECT use is recommended for adolescents with serious psychiatric disorders, such as persistent major depression, schizoaffective disorder, schizophrenia, or history of manic episodes, with or without psychotic features [19]. Other studies point out that ECT can be used in this population also to treat catatonia and neuroleptic malignant syndrome [26,30]. Still according to AACAP's guideline, patient's symptoms must be severe, persistent, and significantly disabling, what can include life-threatening symptoms, such as refusal to drink or eat, uncontrollable mania, florid psychosis, and severe suicide intentions [27]. The lack of treatment response - when considered at least two adequate trials of correct drugs, in association with other therapeutic modalities - is also one of the three criteria, and the fulfillment of this criterion may require patient's observation in a hospital setting [20].

Electroconvulsive therapy may be considered earlier in cases when psychopharmacological treatment is not tolerated by the patient, when adolescent is significantly incapacitated, not being able to take medication, or when waiting for response of psychopharmacological treatment may put the patient's life at risk [17,27].

After conducting a thorough medical evaluation, the psychiatrist decides whether the patient is to be treated with ECT. The severity of illness must be evaluated with extra care by a detailed patient interview, as well as by gathering information from parents or caregivers and by the use of a reliable symptom assessment scale [38]. It is also important to know about previous pharmacological treatment (asking about drugs, dosage, duration, compliance, response, and side effects). In some cases, compliance rate evaluation requires measuring urine and serum drug levels. Psychotherapeutic approaches (individual, interpersonal, or cognitive-behavioral therapies) experienced by the patient should also be recorded in the patient's medical chart.

Despite being as effective in adolescents as it is in adults [29], ECT is much less frequently used in adolescents. Three of the retrieved studies compared ECT use in adolescents with ECT use in patients of other ages. Adolescents subjected to ECT accounted for only 0.43% of the total in India, 0.93% in Australia, and 1.5% in the USA [32,33,36].

### Electroconvulsive therapy treatment parameters

According to the technique of choice, electroconvulsive therapy may alter its efficacy. The study of Antunes and colleagues [15] showed that high-dose unilateral ECT sessions have equivalent efficacy as bifrontotemporal ECT; on the other hand, low-dose unilateral ECT has lower efficacy. The same research also brings preliminary results of studies that suggest that right unilateral ECT with ultra-brief stimulation, achieved by modern ECT equipment, preserves the efficacy and substantially reduces the cognitive side effects.

Another study [27] states that the use of unilateral electrode placement applied to the non-dominant cerebral hemisphere (usually the right brain in most right-handed persons) is associated with less memory impairment in the immediate post-treatment period, recommending bilateral treatment only if unilateral treatment does not reach an adequate response.

Still regarding electrode placement, Shoira and Hamoda [13] state that although bilateral electrode placement may be more effective than unilateral placement for manic patients, unilateral electrode placement has been found to have equivalent results for other indications. However, higher stimulus doses are needed in unilateral treatment to achieve comparable efficiency. Unilateral stimulation at six times the seizure threshold was found to have a remission rate of 55%, compared with 61% for bifrontal stimulation and to 64% for bitemporal stimulation at 1.5 times the seizure threshold. When employing ultra-brief stimulation with modern ECT equipment, unilateral electrode placement at six times the seizure threshold has even been reported to be more effective than bilateral placement at 1.5 times the seizure threshold.

Summing up, it can be speculated that the best technique of ECT is unilateral, over the non-dominant cerebral hemisphere, using modern equipment capable of brief or ultra-brief stimulation. A study with a quantitative approach, comparing the efficacy and side effects of unilateral and bilateral approaches, would be extremely helpful to patients and procedure guidance.

Baeza and colleagues [7], on a study with a sample of 13 adolescents, used the bifrontotemporal ECT until the acute symptoms remitted or no further improvement was shown over three consecutive sessions. Positive results appeared within 13.9 ± 4.3 ECT sessions, with a mean duration of electroencephalogram seizures of 43.9 ± 16.9 s (range, 20 to 93), with significant differences between males and females. Baghai and Moller [16] state that the remission rate of psychotic depression approaches 90%, with relief experienced within 10 to 14 days; other acute psychiatric syndromes such as severe excitement, e.g., in delirious mania, malignant catatonia, and neuroleptic malignant syndrome, may require ECT as a first-line treatment.

### Efficiency of remission, association with other therapies, and comparison between techniques

Several trials have investigated remission as a parameter of efficiency of ECT use in adolescents. A study comparing patients who accepted ECT with those who refused this treatment showed that the first group improved significantly compared to the second [19]. The patients treated with ECT also had a shorter mean duration of hospital stay (73.8 vs. 176 days) [27]. In ten adolescents with primary, endogenous, psychotic depression who were resistant to antidepressant pharmacotherapy, ECT resulted in 60% of complete remission and 40% of partial remission after 1 month post-ECT [10].

In a cohort study with a sample that included adolescents, remission rates after treatment with ECT was equal for both adolescents and adults (58% of remission). The difference between the two studied groups was that most adolescents had psychotic syndromes, while adults had mainly affective disorders [35]. Maintenance ECT in association with pharmacological treatment presented good outcomes for refractory schizophrenia. ECT sessions in a group with drug-resistant schizophrenia resulted in an improvement in quality of life [27,32].

### Side effects, risks, and complications of ECT use

In 1990, the APA Task Force on ECT cited no absolute contraindications to ECT [38]. Although some studies claim that even patients with severe cardiovascular conditions or neurological diseases may benefit from this therapy, guidelines still consider that more studies are needed to allow ECT use in such medical conditions [24,25]. Central nervous system tumors with high levels of cerebrospinal fluid, active pneumonia, and recent myocardial infarction are commonly considered relative contraindications in adolescents [39].

After an ECT session, patients must have their vital signs monitored for 1 to 2 h. Delayed seizures are infrequent but can occur until 48 h after the procedure, representing a reason for monitoring patients in this period [37].

Tardive or prolonged seizures and memory impairment are adverse effects commonly associated to electroconvulsive therapy [39]. The health care team must be attentive to potential harmful effects of general anesthesia [31]. Headache, nausea and vomiting, agitation, and mental confusion are often reported as the most common adverse effects of ECT [14].

### Limitations of the retrieved studies

Finally, it is important to highlight the methodological characteristics of the studies described in the present review, as methodological differences hamper the comparability and generalization of the results. The small samples with which most studies dealt with [7,12,17,18,21,29,31,34], although probably caused by ethical barriers concerning experimental studies involving adolescents, reinforce the importance of assessing the consistency of the results found.

There were also other methodological limitations, such as the absence of control groups [7,30]. Although authors claim that these peculiarities do not affect the results, the generalization and reproducibility of findings may be impaired.

## Conclusions

The common-sense knowledge that ECT is a risky practice makes it the most controversial and polemic treatment approach in psychiatry. However, even after the development of psychopharmacology in the 1950s, ECT maintains its relevance for psychiatry. Nowadays, ECT is still considered a highly efficient option for treatment of several psychiatric disorders, achieving high rates of remission, with few and relatively benign adverse effects.

Research on the use of ECT in adolescents can be considered recent, since the first studies date from the 1980s. Despite of that, ECT is the treatment of choice depending on diagnosis, severity of symptoms, and lack of response to psychopharmacotherapy. The majority of the studies in the scientific literature show the efficiency of ECT use in adolescents and consider this approach more efficient than psychopharmacotherapy isolated.

If ECT is performed in agreement with the guidelines, risks are relatively low. In addition, an experienced staff and adequate physical conditions can minimize the risk of complications. Taking this into account, an important area that requires further research is the development of programs that successfully prepare health care professionals to deal with ECT in the clinical ambience, being able to adequately conduct ECT procedures so as to stop the deleterious effects of the psychopathologies.

## Competing interests

The authors declare that they have no competing interests.

## Authors’ contributions

MLRN, AOAR, NNRL, VBN, JCA, and CACV conceived of the study and participated in its design and coordination. JACP, MMM, SAT, JGJ, FTCJ, and DDMG drafted the manuscript. FTCJ, DDMG, AQB, JSC, and MA revised the manuscript. MLRN, AQB, JSC, and MA revised it critically for important intellectual content. All authors read and approved the final manuscript.
